# Syntheses and Characteristics of Urushiol-Based Waterborne UV-Cured Wood Coatings

**DOI:** 10.3390/polym13224005

**Published:** 2021-11-19

**Authors:** Chia-Wei Chang, Jyun-Ya Liao, Kun-Tsung Lu

**Affiliations:** Department of Forestry, National Chung Hsing University, 145 Xingda Rd, Taichung 402, Taiwan; dimmerc@hotmail.com (C.-W.C.); bear44451@gmail.com (J.-Y.L.)

**Keywords:** oriental lacquer, urushiol, epoxide urushiol, acrylic epoxide urushiol, WUV wood coatings

## Abstract

The manufacture and properties of waterborne UV-cured coatings (WUV coatings) by acetone process based on urushiol for wood finishing were investigated. Firstly, epoxide urushiol (EU) was prepared by reacting urushiol with epichlorohydrin. Secondly, the EU was reacted with acrylic acid to obtain acrylic epoxide urushiol (AEU). Next, the prepolymers were synthesized by the reaction of AEU, 2,2-Bis(hydroxymethyl)propionic acid (DMPA), and isophorone diisocyanate (IPDI) and hexamethylene diisocyanate (HDI), respectively, using acetone as a solvent. The prepolymers were further neutralized by triethylamine (TEA) to obtain ionomers and dispersed in the water. After removing the acetone by vacuum distillation, the polyurethane dispersions (PUDs) were obtained. Finally, the WUV coatings were performed by adding a photoinitiator (Irgacure 2959). The products in the synthesized processes and the properties of the WUV coatings were examined. The results showed that the EU, AEU, prepolymers, and ionomers could be synthesized stably. The PUDs synthesized by the IPDI and HDI had a similar solid content of 25.2% and 26.2%, and similar pH values of 7.8 and 7.6. However, the IPDI-containing PUD displayed lower viscosity, smaller particle size, and a more even polydispersity index. The IPDI-containing WUV film displayed a higher hardness, gloss, and lightfastness. The HDI-containing WUV film possessed superior impact resistance. Both IPDI-containing and HDI-containing WUV films showed excellent adhesion, bending resistance, and mass retention, and demonstrated a potential for wood finishing.

## 1. Introduction

In recent decades, excessive reliance on petrochemicals has resulted in irreversible environmental pollution problems, including the greenhouse effect, air pollution, unrecyclable garbage, etc., and these forms of pollution have directly or indirectly caused risks to human health. Furthermore, high costs and carbon emissions trading also promoted the partial or complete substitution of petrochemicals with renewable biomass [1,2,3,4]. Raw oriental lacquer is a natural and renewable polymeric material with water in oil (W/O) emulsion sap, which is obtained by tapping *Rhus* trees in Southeast Asia [5,6,7]. The global lacquer sap output is over 3800 ton in the year 2014, and the lacquer tree cultivation area in Asia increases every year. In Vietnam alone, the lacquer sap output had increased from 46 tons in the year 2000 to 350 tons in the year 2015. [8]

The components of raw oriental lacquer are 50–65% of catechol derivatives such as urushiol, 20–30% of water, 5–7% of plant gum (polysaccharides), 2–5% of nitrogenous compounds, and approximately 1% of laccase [9,10,11,12]. Its film possesses a wax-like gloss, an elegant appearance, and high durability compared to synthetic coatings and is widely used for wood furniture and handicrafts finishing.

The urushiol, the main component of raw oriental lacquer, consists of 3-substituted catechol derivatives with 0–3 olefins in the side chain [13,14,15,16], which features a unique chemical structure and complex reactive groups, including hydroxyl, phenol, catechol, and unconjugated and conjugated double bonds. These functional groups lead to a variety of reactive possibilities [17,18,19,20,21,22,23] and the highly diverse utilization of urushiol as a bio-material.

In this study, the urushiol was extracted from oriental lacquer sap using acetone as a solvent and applied to the manufacture of waterborne ultraviolet cured coatings (WUV coatings) for wood finishing. Traditional UV coating offers some advantages such as fast curing, lower energy consumption, high production, superior quality of film properties, space-saving, and suitability for wood finishing. However, they also feature poor adhesion and cause irritation. However, WUV coatings have no toxicity or odors, which derive from the monomers on traditional UV coatings [24,25]. Futhermore, they possess many merits, including the advantages of traditional UV coating mentioned above, low toxicity, low flammability, water-reducibility, and low numbers of volatile organic compounds (VOCs); they are suggested for applyication in the coating industry [26,27,28,29].

In this study, two kinds of urushiol-based WUV coatings with isophorone diisocyanate (IPDI) and hexamethylene diisocyanate (HDI) were synthesized by using the acetone process. The fundamental properties of WUV films were also examined to explore their application on wood finishing. We expect that the film adhesion can be improved through the additional ductile side chains of the urushiol in the film.

## 2. Materials and Methods

### 2.1. Materials

The oriental lacquer was collected from the cultivar *Rhus succedanea* and was purchased from the Long-Nan Museum of Natural Lacquer Ware (Nantou, Taiwan). The epichlorohydrin was provided by Alfa Aesar (Haverhill, MA, USA). The sodium hydroxide (NaOH) and acetone were obtained from Union Chemical Works Ltd. (Hsinchu, Taiwan). The benzyltriethylammonium chloride was purchased from Acros Organics (Waltham, MA, USA). The acrylic acid was supplied by Hayashi Junyaku Kogyo Co., Ltd. (Kyoto, Japan). The triethylamine (TEA) was obtained from Nippon Bacterial Test Co., Ltd. (Osaka, Japan). The IPDI, HDI, 2,2-Bis (hydroxymethyl)propionic acid (DMPA), and a photoinitiator, 2-Hydroxy-1-[4-(2-hydroxyethoxy)phenyl]-2-methyl-1-propanone (Irgacure 2959), were provided from Merck (Darmstadt, Germany). The dibutyltin dilaurate was obtained from Sigma-Aldrich (SL, USA). The *Cunninghamia lanceolate* wood specimens with a size of 8 cm (R) × 15 cm (L) × 1 cm (T) were sanded by the sequence of #180 and #400 sandpaper. Glass plates with a size of 15 cm × 10 cm × 0.2 cm, polyethylene terephthalate (PET) plates, and tin-coated iron sheets (20 cm × 5 cm × 0.05 cm) (Sheng Huei Instrument Corp., Taichung, Taiwan) were prepared as the experimental substrates, as specified by the CNS 9007 Standard [30].

### 2.2. Preparation and Measurement of Epoxide Urushiol (EU) and Acrylic Epoxide Urushiol (AEU)

**Figure 1:**
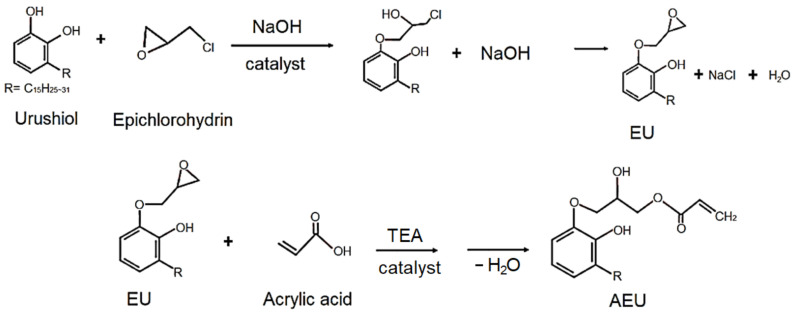
Syntheses of the epoxide urushiol (EU) and acrylic epoxide urushiol (AEU).

The urushiol was extracted from the oriental lacquer using the acetone as a solvent, and then the acetone was removed by vacuum distillation. The 100 g urushiol was mixed with epichlorohydrin with a urushiol/epichlorohydrin mole ratio of 0.0625, and 5 g benzyltriethylammonium chloride was added as a catalyst. The reaction was carried out at 60 °C for 3 h, and 60 g of 50% NaOH _(aq)_ was added dropwise in the following 3 h. Next, the reaction was continued and kept at 60 °C for another 1 h. The mixture was cooled to room temperature and the acetone was added to purify and precipitate the generated NaCl. Finally, the excess of epichlorohydrin and acetone was removed by vacuum distillation and the EU was obtained. Subsequently, the EU was mixed with the acrylic acid with a COOH/epoxide mole ratio of 1, and 1 phr TEA was added as a catalyst. The reaction was maintained at 80 °C for 5 h. After cooling the mixture at room temperature, the water was removed by vacuum distillation, and the AEU was obtained. The synthesized process is drawn in Figure 1.

The Fourier-transform infrared spectroscopy (FTIR) analyses of the EU and AEU were performed by a transmission method with a Perkin–Elmer spectrum 100 (Perkin Elmer, Shelton, CT, USA). The hydrogen nuclear magnetic resonance (^1^H NMR) analyses of EU and AEU were acquired using Agilent Technologies DD2 600 (Agilent Technologies, Santa Clara, CA, USA). To determine the epoxy equivalent weight (EEW) of the EU, epoxy titration was performed according to the ASTM D1652-90 [31]. The hydroxyl value (HV) of the AEU was conducted according to CNS13568 K0058 [32].

### 2.3. Preparation and Measurement of Urushiol-Based Polyurethane Dispersions (PUDs) with Different Diisocyanates

Firstly, the DMPA was mixed with different diisocyanates, HDI and IPDI, respectively, with an NCO/OH mole ratio of 0.53, and placed in a four-neck reaction flask. The mixture was then diluted to 50 wt.% by adding acetone and reacted in the nitrogen atmosphere at 60 °C for 1 h. After the NCO content of the mixture was decreased to 50% of its initial NCO content, the calculated weight of the AEU was added. The temperature of the reaction was kept at 60 °C for another 6 h, and the carboxyl-containing prepolymers with different diisocyanates were obtained.

**Figure 2:**
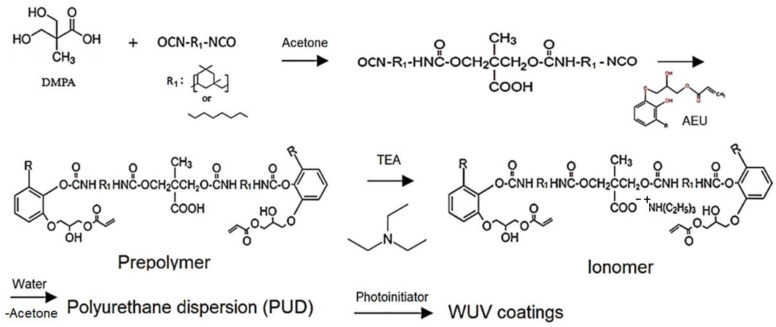
Manufacture of the urushiol-based WUV coatings.

Secondly, the carboxyl-containing prepolymers were mixed with TEA at a COOH/TEA mole ratio of 1 under 200 rpm stirring for 30 min, and the ionomers with R-COO^− +^N-R groups were obtained. Subsequently, acetone was added to adjust the solid content of the ionomer to 50 wt.%, and then the deionized water was added dropwise at a rate of 4 mL/min into the ionomer with a stirred rate of 600 rpm. During the process of phase transition, the viscosity change of the dispersions was used to speculate the phase inversion point, which is the transition of the dispersed phase from the oil to water phase. After that, the acetone was removed by vacuum distillation and the PUDs were obtained. The synthesized processes are shown in Figure 2.

The molecular weight of the prepolymer was conducted by gel permeation chromatography (GPC, Hitachi-L6200, Hitachi High-Tech Fielding Corp., Tokyo, Japan) with a Shodex KF-802.5 column (Showa Denko K.K., Tokyo, Japan) and Hitachi L-4000 UV detector (Hitachi High-Tech Fielding Corp., Tokyo, Japan) at a detection wavelength of 254 nm. The viscosity of the ionomers and PUDs were measured by a Brookfield viscosimeter DV-E (Brookfield Engineering, Middleboro, USA) with a C50-1 cone spindle at 25 °C and a fixed shear rate of 300 s^−1^. The solid content of the PUDs was estimated in accordance with CNS 5133 [33]. The pH value of the AEU was measured by Suntex sp-701 (Suntex Instruments Co., Ltd., New Taipei City, Taiwan) at 25 °C. The particle size and the polydispersity index (PDI) of the PUDs were measured by dynamic light scattering (Malvern Nano-ZS, MicrotracBEL Corp., Osaka, Japan) equipped with laser diffraction and detectors (detected range 0.6–6000 nm). The stability of the PUDs was performed by storing the PUDs in the polyethylene bottle, and its changes in appearance, including precipitation, separation, gelling, and discoloration, were observed.

### 2.4. Preparation of The Urushiol-Based WUV Coatings

#### 2.4.1. Curing Processes of WUV Coatings

The 3 wt.% photoinitiator, Irgacure2959, was added to the PUDs and the WUV coatings were obtained. The viscosity of the WUV coatings was adjusted to 2000 cps by adding water and applied onto the substrates by a film applicator (Elcometer Co., Manchester, UK) with a wet-film thickness of 100 μm. All the specimens were placed at room temperature for 10 min and then transferred to a 50 °C oven for another 5 min. The specimens were cured using UV equipment (UVC-362W, C-SUN, Taiwan) with both a mercury lamp (the main wavenumber was 365 nm) and a halogen lamp (the main wavenumber was 420 nm). The radiation distance was 10 cm, and the conveyor speed was 8 m/min; the curing process was repeated three times, which corresponded to an irradiation time of 18 s. All of the specimens were placed in a 25 °C and 75%RH environment for 7 days and then the film properties were measured.

#### 2.4.2. Measurements of WUV Film Properties

The hardness of the film on the glass plate was measured by a König/Persoz pendulum hardness tester (Braive Instruments, Liège, Belgium) according to the DIN 53,157 with the repeated number of 10. In the test of the film mass retention, free films were separated from the PET substrates and soaked in a Soxhlet extractor (Merck, Darmstadt, Germany) with refluxing acetone for four cycles (fill/siphon)/h. After 6 h of extraction, the soaked films were oven-dried at 50 °C for 6 h and their mass retentions were calculated. The impact resistance was measured by an impact tester (IM-601, IDM Instruments Pty Ltd., Hallam, Victoria, Australia). The impact needle featured a diameter of 1/2 inch and the falling hammer weighted 300 g. The height of intact film was recorded. The film adhesion was measured by the crosscut method outlined in CNS 10,756 K 6800 [34]. The adhesions of the film, from the best to the worst, were 10, 8, 6, 4, 2, and 0. The bending resistance was tested using the film finished on the tin-coated iron according to JIS-K-5400 [29] by a bending tester (Ueshima Seisakusho Co., Ltd., Tokyo, Japan) with steel bar diameters of 2, 3, 4, 6, 8, and 10 mm. The film passed the test of smaller diameter bars demonstrated better bending resistance. The film gloss on the glass plate was detected by a Dr. Lange 20° Reflectometer (Dr. Bruno Lange GmbH, Berlin, Germany) Fifteen points were measured and averaged for each specimen according to JIS K 5400 [35]. The film’s lightfastness was measured by a Paint Coating Fade Meter (Suga Test Instruments, Tokyo, Japan). The films were irradiated by mercury light (H400-F) at a chamber temperature of 32 ± 5 °C for 200 h. The color changes of the films were measured by a spectrophotometer (CM-3600d, Minolta, Osaka, Japan) with an 8 mm target mask and fitted with a D65 light source with a measuring angle of 10°. The color difference (ΔE^*^) and the yellow index difference (ΔYI) in CIE L∗a∗b∗ system were used to evaluate the lightfastness of the films.

## 3. Results and Discussion

### 3.1. Structure Identification and Characteristics of EU and AEU

**Figure 3:**
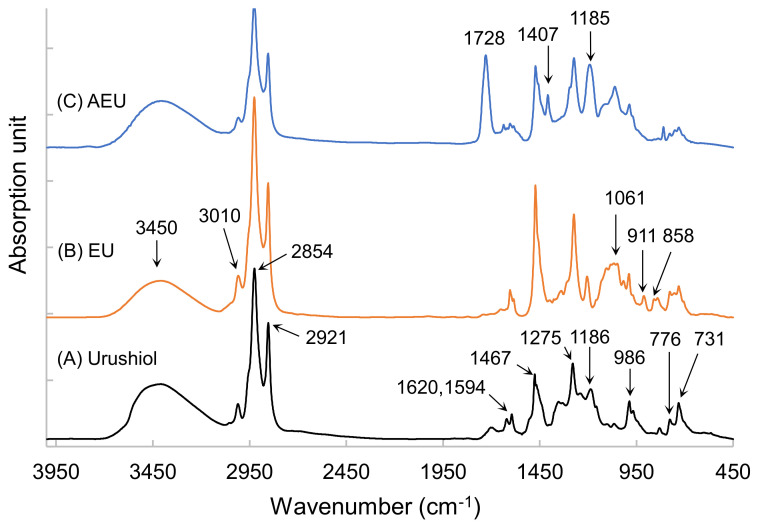
FTIR spectra of (**A**) urushiol, (**B**) EU, and (**C**) AEU.

To further confirm the synthetic products, the FTIR analyses were used to detect the changes in the functional groups among the urushiol, EU, and AEU. Their spectra are drawn in Figure 3. In the urushiol spectrum, the absorptions at 3150–3600 cm^−1^ and 1275 cm^−1^ represented the stretching and bending vibrations of the phenol group, respectively. The peaks at 776 cm^−1^ and 731 cm^−1^ were associated with the benzene ring of the urushiol, and the peaks at 1620 cm^−1^ and 1594 cm^−1^ represented the C=C stretching vibration in the benzene ring of the urushiol. The alkyl side chain linked to the catechol through ether linkage demonstrated an absorption peak at 1185 cm^−1^ [27]. The C–H stretching vibration of the unsaturated double bond demonstrated a peak at 3010 cm^−1^, and the conjugated C=C of the urushiol side chain was observed at 986 cm^−1^. The peaks at 2921 cm^−1^ and 2854 cm^−1^ represented the asymmetrical and symmetrical vibrations of methylene (–CH_2_–), and the bending vibration of the methylene was also found at 1467 cm^−1^.

After the reaction of the epichlorohydrin and the urushiol, the peaks appeared at 858 cm^−1^ and 911 cm^−1^, which related to the epoxide group in the EU spectrum (as shown in Figure 3B). The peak at 1061 cm^−1^ represented the C–O linkages. Besides, the other peaks were similar to that of urushiol’s spectrum. These results confirmed that the epoxide group was introduced into the EU molecule.

The AEU was generated through the reaction of the EU and the acrylic acid. The epoxide group was consumed in the reaction with the –COOH group. Therefore, the peaks of epoxide at 858 cm^−1^and 911 cm^−1^ decreased, and the new peaks at 1728 cm^−1^ (–C=O), 1407 cm^−1^ (COO), and 1061 cm^−1^ (C–O) appeared (as shown in Figure 3C). The peak intensity at 1185 cm^−1^ also increased, which resulted from the generation of ether groups from the ring-opening reaction. These results showed that the AEU can be synthesized stably.

**Figure 4:**
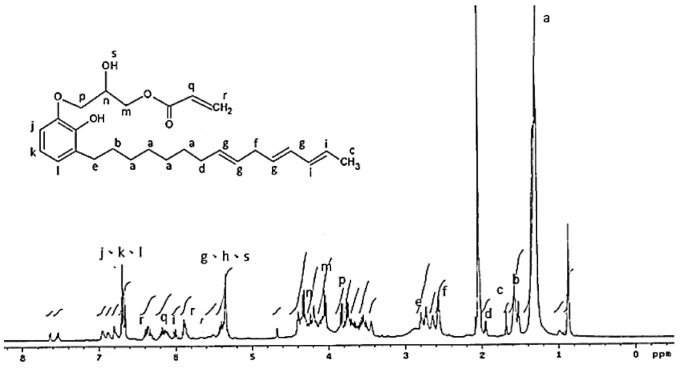
^1^H NMR spectrum of AEU.

The ^1^H NMR analysis of the AEU is drawn in Figure 4. The signals at 0.70–3.00 ppm were assigned to the protons in the saturated alkyl group of the AEU side chain. Among these ranges, the signal at 1.29–1.35 ppm (a) represented the protons of methylene (–CH_2_–), and the signal at 1.59 ppm (b) was the signal of the protons in the second methylene linked to the phenol (Ph–CH_2_–CH_2_–). The signal at 1.67 ppm (c) was generated from the protons of the terminal methyl group. The peak at 1.97 ppm (d) was assigned to the protons in the unsaturated double bond (–CH_2_–CH=). The peak at 2.80 ppm (e) was the signal of methylene linked to the phenol (Ph–CH_2_–). The peaks at 2.59–2.61 ppm (f) were assigned to the unconjugated double bond (=CH–CH_2_–CH=). The peaks at 6.55–6.59 ppm (j, k, l) were associated with the benzene ring, and the peaks at 5.98–6.05 ppm (i) were assigned to the conjugated double bond (–CH=CH–CH=CH–). These results showed that the main structure and specific groups of the urushiol were retained. In addition, the signal at 4.02 ppm (m) was the generated methylene protons after the ring-opening reaction. The peaks at 5.31–5.39 ppm (g) were assigned to the protons in the double bonds of the side chains (–CH=CH–), phenols (h), and protons in the generated hydroxyl groups after the ring-opening reaction (–OH) (s). The results mentioned above confirmed that the epoxide reaction of urushiol and epichlorohydrin progressed well. Furthermore, the peaks at 6.15–6.19 ppm (q) were associated with the proton of the acryl group (O=C–CH=), and the peaks at 5.89–5.91 and 6.33–6.40 ppm (r) were assigned to the terminal protons of the acryl groups (O=C–CH=CH**_2_**) [36,37,38,39]. These results revealed that the terminal double bonds derived from acrylic acid were introduced into the AEU molecule successfully.

**Figure 5:**
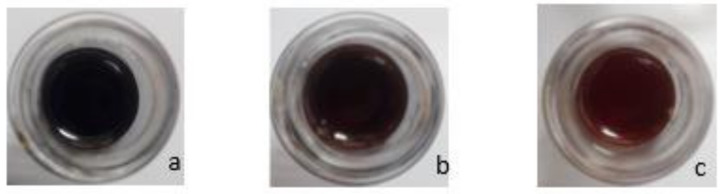
Appearances of (**a**) urushiol, (**b**) EU, and (**c**) AEU.

The appearances of the urushiol, EU, and AEU are listed in Figure 5. The urushiol had a black color and the epoxy equivalent weight (EEW) was 0 g/eq. After the epoxide reaction, the EU transferred to a brown color demonstrated an EEW of 380 g/eq, which indicated that each EU molecule possessed approximately one epoxide group (the theoretical molecular weight of EU is 373 g/mole). The EU was then reacted with acrylic acid, and the AEU was generated with a light brown color, a hydroxyl value (HV) of 253 mgKOH/g, and an EEW of 11,554 g/eq. The huge EEW value of the AEU revealed that there was almost no epoxide group in the AEU molecule. Furthermore, the AEU demonstrated a similar HV value to the theoretical value of 259 mgKOH/g. The results also demonstrated the AEU could be synthesized effectively.

### 3.2. Structure Identification and Molecular Weight of Prepolymers

**Figure 6:**
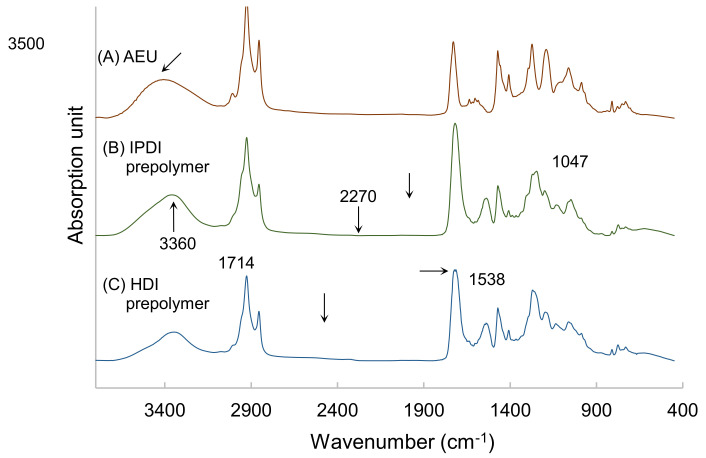
FTIR spectra of (**A**) AEU, (**B**) IPDI-containing prepolymer, and (**C**) HDI-containing prepolymer.

The prepolymers synthesized by HDI and IPDI, respectively, were analyzed by FTIR, and the results are drawn in Figure 6. Both of the spectra of IPDI-containing and HDI-containing prepolymers were similar. The peak at 3500 cm^−1^ was associated with the –OH group of the AEU, and this peak shifted to the 3360 cm^−1^, which represented the stretching vibrations of the N–H groups in the urethane structures of the IPDI-containing and HDI-containing prepolymers. In addition, the N-H bending vibration at 1538 cm^−1^, the C=O stretching vibration at 1714 cm^−1^, and the C–O stretching vibration at 1047 cm^−1^ were found in the spectra. These results also confirmed that the urethane structures were determined in the films [40,41]. Furthermore, the peaks of the –NCO group, which was usually found at 2270 cm^−1^, disappeared in both the spectra of the IPDI-containing and the HDI-containing films. This result proved that the –NCO groups were consumed completely in the syntheses of IPDI-containing and HDI-containing prepolymers.

**Figure 7:**
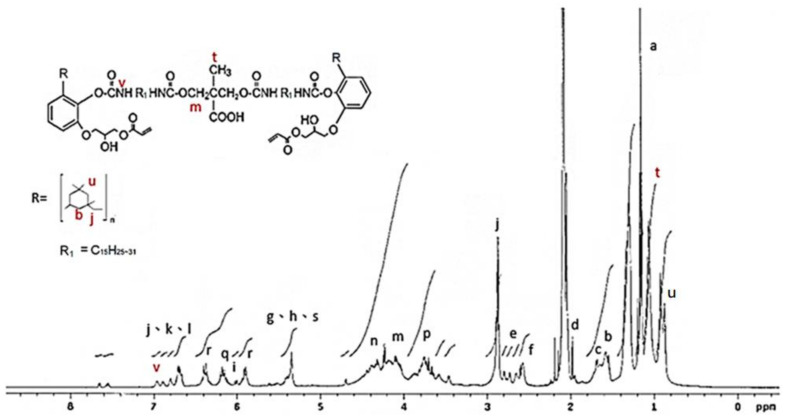
^1^H NMR spectrum of IPDI-containing prepolymer.

**Figure 8:**
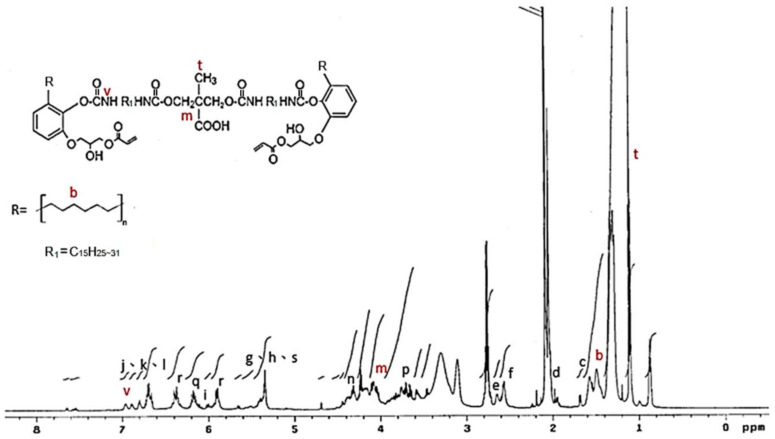
^1^H NMR spectrum of HDI-containing prepolymer.

The ^1^H NMR spectrum of the IPDI-containing prepolymer is drawn in Figure 7. The signal at 0.8 ppm and 1.1 ppm represented the –C**H**_3_ in the structures of IPDI (u) and DMPA (t) [42]. The methylene (–C**H**_2_–) was recorded at 1.3–1.9 ppm (b), and the signal of the methylene linked to the nitrogen atom (–C**H**_2_–HN–) was at 2.8 ppm (j). The peak at 4.0–4.44 ppm (m) was the protons of the –O–C**H**_2_- structures of the DMPA and the peak at 6.8 ppm (v) was associated with the protons in the urethane structures (–N**H**–). The ^1^H NMR spectrum of HDI-containing prepolymer (as shown in Figure 8) was simpler than that of IPDI-containing prepolymer due to the lack of cyclic aliphatic structures. The FTIR and the ^1^H NMR analyses showed that both the HDI-containing and IPDI-containing prepolymers can be synthesized readily and successfully.

**Figure 9:**
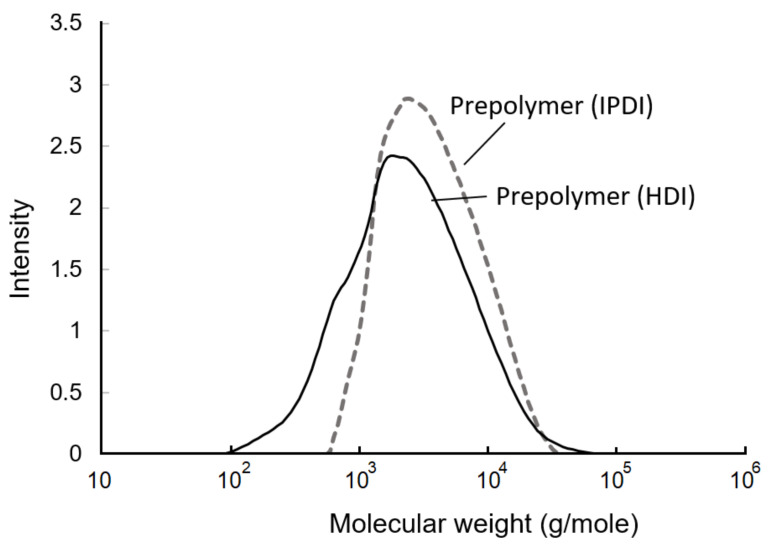
Gel permeation chromatography (GPC) curves of prepolymers.

The GPC curves of the prepolymers are shown in Figure 9. Based on the calculations, the weight-average molecular weight (Mw), number-average molecular weight (Mn), and polydispersity are listed in Table 1. The Mw, Mn, and polydispersity of te IPDI-containing prepolymer were 4583 g/mol, 2535 g/mol, and 1.8, respectively. In the HDI-containing prepolymer, these values were 3657 g/mol, 1390 g/mol, and 2.6, respectively. The fact that the molecular weight of the HDI-containing prepolymer was lower than that of IPDI-containing prepolymer may have been due to the higher and more even reactivity of the NCO groups of HDI than those of IPDI, resulting in the observation of a fast additive reaction and a broader molecule distribution. Moreover, the primary NCO group demonstrated a lower reactivity compared to the secondary NCO group in the IPDI. Therefore, the additive reaction with the IPDI trended to the step-reaction polymerization, and resulted in a narrow molecule distribution [43], which was also confirmed with the HDI-containing prepolymer displaying a larger polydispersity of 2.6 than the IPDI-containing prepolymer, whose value was 1.8.

### 3.3. Fundamental Properties of PUDs

**Figure 10:**
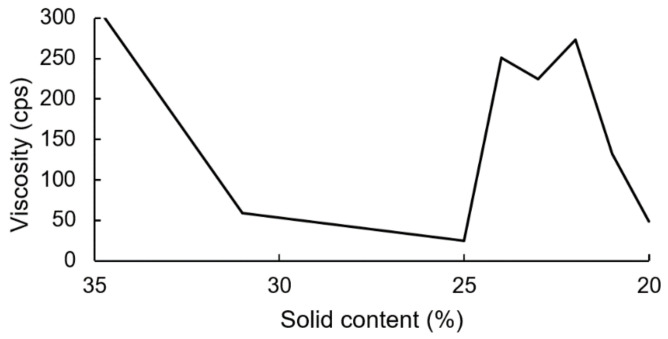
Viscosity variations of IPDI-containing ionomer during water dispersion process.

**Figure 11:**
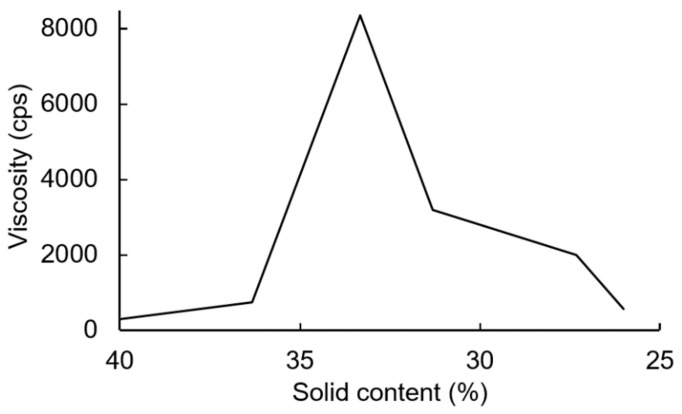
Viscosity variations of HDI-containing ionomer during water dispersion process.

The PUDs were synthesized firstly by neutralizing carboxyl-containing prepolymers with TEA, and ionomers were obtained. Next, the ionomers were dispersed through the addition of deionized water. The phase inversion point during the water dispersion process was determined by the variations in the viscosity. The viscosity variation of the water–acetone dispersions of the IPDI-containing ionomer in Figure 10 showed that the phase inversion points occurred within a solid content range of 23–24%. When the solid content of the dispersion reached 20%, large viscosity variations were no longer observed. Therefore, a solid content of 20% was considered the endpoint of the phase inversion. Figure 11 shows the viscosity variations during the water dispersion process of HDI-containing ionomer. The phase inversion point occurred within a solid content range of 30–35%. When the solid content of the dispersion reached 26%, the viscosity was not changed, which was also considered the endpoint of the phase inversion. The results also showed that when the dispersion reached the phase inversion point, the HDI-containing ionomer dispersion demonstrated a higher viscosity than the IPDI-containing ionomer dispersion. This phenomenon is attributed to the alkyl chain entanglement of the HDI.

**Figure 12:**
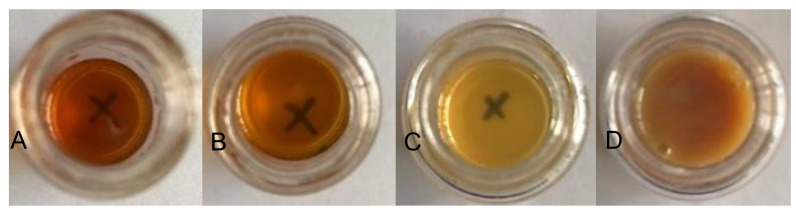
Appearances of prepolymers synthesized with (**A**) IPDI, (**B**) HDI, and of PUDs synthesized with (**C**) IPDI and (**D**) HDI.

**Figure 13:**
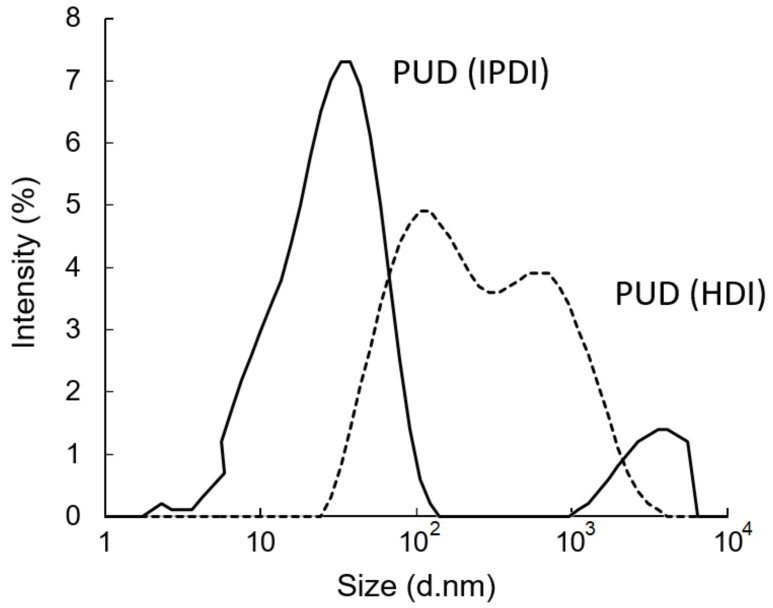
Particle size distributions of PUDs.

After removing the acetone from the water–acetone dispersions by vacuum distillation, the polyurethane dispersions (PUDs) were obtained. The appearances of the prepolymers and PUDs, which were synthesized from the HDI and IPDI, respectively, are listed in Figure 12. The appearance of the IPDI-containing prepolymer was light-brown in color (A), and the IPDI-containing PUD displayed a semitransparent and light-yellow color (C). The HDI-containing prepolymer also displayed a light-brown color (B), while the appearance of the HDI-containing PUD was opaque and of a milky-brown color (D). These phenomena can be attributed to their fundamental properties, which are listed in Table 2. Although the IPDI-containing and HDI-containing PUDs demonstrated similar solid contents of 25.2% and 26.2%, and similar pH values of 7.8 and 7.6, the obvious differences were found in the viscosity and particle size of the PUDs. The IPDI-containing PUD featured a smaller z-average particle size of 80 nm, and its PDI was a narrow distribution of 0.217. By contrast, the HDI-containing PUD demonstrated a larger z-average particle size of 158 nm and a PDI of 0.524, indicating a more uneven particle composition than the IPDI-containing PUD. The particle size distributions of the PUDs measured by dynamic light scattering (DLS) are shown in Figure 13. The appearances of the waterborne dispersions are associated with their particle size. When the WPU has a smaller particle size, a more transparent appearance is observed [25,44]. In this study, the HDI-containing PUD had a particle size of 158 nm and an opaque and milky-brown color, while the IPDI-containing PUD was 80 nm, and a semitransparent and light-yellow color was obtained. In a study by Ley et al. [45], waterborne coatings prepared by different acrylic acids featured different particle sizes, from 145 to 177 nm. Nanda et al. [46] found that the particle sizes of their WPU coatings are relatively small, from 40 to 140 nm, according to the differences between the components. In this study, the particle sizes of IPDI-containing and HDI-containing PUDs were also confirmed, in line with those reported previously.

The IPDI-containing PUD demonstrated a viscosity of 100 cps and no precipitation or separation was observed after 1 month at room temperature, indicatinthat excellent stability of the IPDI-containing PUD was achieved. Comparatively, the HDI-containing PUD demonstrated a higher viscosity of 2000 cps and slight precipitation occurred after 1 month, deriving from larger particles of the HDI-containing PUD have strong interactions and gravitation, and particles are easy to deform, merge and result in precipitation and separation [47].

### 3.4. Fundamental Properties of WUV Films

The 3 wt.% photoinitiator, Irgacure2959, was added to the PUDs and the waterborne UV-cured coatings (WUV coatings) were obtained. After curing by UV irradiation, the fundamental properties of the WUV films are listed in Table 3. The IPDI-containing WUV film had a higher hardness of 131 sec than the HDI-containing WUV film of 41 s. The IPDI-containing and HDI-containing WUV films featured similar mass retention of 53% and 51%, respectively. These results showed that the higher hardness of the IPDI-containing WUV film was attributed to the cyclic aliphatic structures in the IPDI diisocyanate. In addition, the HDI-containing WUV film demonstrated a better impact resistance, of 10 cm, than the IPDI-containing WUV film, of 5 cm, due to the long alkyl chains in the HDI diisocyanate. Both the IPDI-containing and HDI-containing WUV films showed a superior adhesion of grade 8, and they also passed the 2 mm test in the bending resistance test. The IPDI-containing and HDI-containing WUV films showed no noticeable difference in the film gloss, but the IPDI-containing WUV film demonstrated a slight increase in the gloss, of 100 GU, than the HDI-containing WUV film, of 84 GU. After 200 h UV irradiation, the IPDI-containing WUV film showed a lower ∆E^*^ and ∆YI of 0.99 and 1.87, receptivity, than the HDI-containing WUV film of 2.00 and 3.93. These results revealed that the IPDI-containing WUV film featured a better lightfastness, which is more suitable for applying on the topcoat coating on wood than that of the HDI-containing WUV coatings.

## 4. Conclusions

In this study, urushiol was used as a bio-material for manufacturing WUV coatings. The results of the EEW determination, FTIR, and ^1^H NMR analyses revealed that the epoxide urushiol (EU) was synthesized successfully, and featured one epoxide group per EU molecule. After the reaction of the acrylic acid and the EU, the acrylic epoxide urushiol (AEU) with the acryloyl (CH_2_=CHCO–) groups was also synthesized readily and successfully. For practical application, the AEU was reacted with two different diisocyanates, IPDI and HDI, respectively, and DMPA to obtain the prepolymers. Next, the prepolymers were neutralized with TEA to obtain ionomers. After water dispersion, the polyurethane dispersions (PUDs) were also synthesized stably. The IPDI-containing and HDI-containing PUDs featured similar solid contents, of 25.2% and 26.2%, and similar pH values, of 7.8 and 7.6. However, the IPDI-containing PUD demonstrated lower viscosity and smaller particle size than the HDI-containing PUD. The IPDI-containing WUV film displayed a higher hardness, of 100 s, and gloss, of 100 GU, than the HDI-containing WUV film. In addition, the HDI-containing WUV film demonstrated a better impact resistance than the IPDI-containing WUV film. Both the IPDI-containing and the HDI-containing WUV films showed superior adhesion on wood and excellent bending resistance. The IPDI-containing film and HDI-containing WUV film alsofeatured similar mass retentions However, the IPDI-containing WUV film showed a better lightfastness than the HDI-containing WUV film

## Figures and Tables

**Table 1:** Molecular weight and polydispersity of prepolymers.

Prepolymers	M_w_ (g/mole)	M_n_ (g/mole)	Polydispersity (M_w_/M_n_)
IPDI	4583	2535	1.8
HDI	3657	1390	2.6

**Table 2:** Fundamental properties of PUDs synthesized with IPDI and HDI.

PUDs	Viscosity (cps)	Solid Content (%)	pH Value	Z-Average Particle Size (nm)	PDI
IPDI	100	25.2	7.8	80	0.217
HDI	2000	26.2	7.6	158	0.524

**Table 3:** Fundamental properties of WUV films synthesized with IPDI and HDI.

WUV Films	Hardness (König, S)	Mass Retention (%)	Impact Resistance (300 g, cm)	Adhesion (Grade)	Bending Resistance (mm)	20° Gloss (GU)	Lightfastness (after 200 h Irradiation)	
							ΔE^*^	ΔYI
IPDI	131 ± 4	53 ± 2	5	8	<2	100 ± 4	0.99	1.87
HDI	41 ± 3	51 ± 5	10	8	<2	84 ± 3	2.00	3.93

## Data Availability

Data available on request from the authors.
